# Machine Learning in Systems Biology

**DOI:** 10.1186/1753-6561-2-s4-s1

**Published:** 2008-12-17

**Authors:** Florence d'Alché-Buc, Louis Wehenkel

**Affiliations:** 1 IBISC CNRS FRE 3190, Université d'Evry-Val d'Essonne, Genopole, Tour Evry II, Evry, France; 2 GIGA-R and Dept. of EE&CS, University of Liège, B4000, Liège, Belgium

## Abstract

This supplement contains extended versions of a selected subset of papers presented at the workshop MLSB 2007, Machine Learning in Systems Biology, Evry, France, from September 24 to 25, 2007.

## Introduction

Molecular biology and also all the biomedical sciences are undergoing a true revolution as a result of the emergence and growing impact of a series of new disciplines/tools sharing the "-omics" suffix in their name. These include in particular genomics, transcriptomics, proteomics and metabolomics devoted respectively to the examination of the entire systems of genes, transcripts, proteins and metabolites present in a given cell or tissue type.

The availability of these new, highly effective tools for biological exploration is dramatically changing the way one performs research in at least two respects. First of all, the amount of available experimental data is not at all a limiting factor any more; on the contrary, there is a plethora of it. The challenge has shifted towards identifying the relevant pieces of information given the question, and how to make sense out of it (a "data mining" issue). Secondly, rather than to focus on components in isolation, we can now try to understand how biological systems behave as the result of the integration and interaction between the individual components that one can now monitor simultaneously (so called "systems biology").

Taking advantage of this wealth of "genomic" information has become a conditio sine qua non for whoever ambitions to remain competitive in molecular biology and more generally in biomedical sciences. Machine learning naturally appears as one of the main drivers of progress in this context, where most of the targets of interest deal with complex structured objects: sequences, 2D and 3D structures, or interaction networks. At the same time bioinformatics and systems biology have already induced significant new developments of general interest in machine learning, for example in the context of learning with structured data, graph inference, semi-supervised learning, system identification, and novel combinations of optimization and learning algorithms.

The aim of the MLSB 2007 workshop on Machine Learning in Systems Biology, held at University of Evry, France, was to contribute to the cross-fertilization between the research in machine learning methods and their applications to complex biological and medical questions by bringing together method developers and experimentalists.

MLSB 2007, was a follow up of the PMSB 2006 workshop on Probabilistic Modeling and Machine Learning in Structural and Systems Biology, held in Tuusula, Finland, from June 17 to 18, 2006 (see also [1]). It has been followed by MLSB 2008, held in Brussels, Belgium, from September 13 to 14, 2008, and will be further followed by MLSB 2009, taking place in Bled, Slovenia, on September 5 to 6, 2009.

## Summary of the supplement

Selected submissions were invited based on the papers presented in the workshop. This supplement contains a reviewed selection of six full papers that cover a large panel of topics in Machine Learning devoted to Systems Biology.

Aastinen et al. [2] develop kernel methods for enzyme function prediction in the framework of structured output prediction methods, where the enzymatic reaction is the combinatorial target object for prediction.

Ying et al. [3] address high throughput analysis of microarray data by using a variational Bayesian inference method for unsupervised clustering that allows latent process variables and model parameters to be dependent.

The work of Omont et al. [4] analyzes genome-wide association studies results of Multiple Scleroris with a new Bayesian model that integrates genotyping errors and genomic structure dependencies.

Azé et al. [5] consider annotation of a protein with terms of the functional hierarchy that has been used to annotate *Bacillus subtilis *and learn a set of rules that predict classes in terms of elements of the functional hierarchy using two methods: first-order and multilabel attribute value decision-trees.

Kontos et al. [6] formulate the identification of putative NCR genes in the yeast *Saccharomyces cerevisiae *as a supervised two-class classification problem and use different classifiers and variable selection methods to predict whether genes are NCR-sensitive or not from a large number of variables related to the GATA motif in the upstream non-coding sequences of the genes.

Birmelé et al. [7] propose to cluster genes by co-regulation rather than by co-expression and propose an inference algorithm for detecting co-regulated groups from gene expression data and then introduce a method to cluster genes given that inferred regulatory structure.
